# Current Practice of High Flow through Nasal Cannula in Exacerbated COPD Patients

**DOI:** 10.3390/healthcare10030536

**Published:** 2022-03-15

**Authors:** Andrea Bruni, Eugenio Garofalo, Daniela Procopio, Silvia Corrado, Antonio Caroleo, Eugenio Biamonte, Corrado Pelaia, Federico Longhini

**Affiliations:** Anesthesia and Intensive Care Unit, Department of Medical and Surgical Sciences, University Hospital Mater Domini, Magna Graecia University, 88100 Catanzaro, Italy; andreabruni87@gmail.com (A.B.); eugenio.garofalo@gmail.com (E.G.); daniela.procopiodoc@libero.it (D.P.); sylviacorrado.sc@gmail.com (S.C.); antonio.caroleo1992@libero.it (A.C.); egbiam@yahoo.it (E.B.); pelaia.corrado@gmail.com (C.P.)

**Keywords:** Chronic Obstructive Pulmonary Disease, oxygen, high flow nasal cannula, non-invasive ventilation, respiratory therapy, respiratory insufficiency, hypercapnia, positive-pressure respiration

## Abstract

Acute Exacerbation of Chronic Obstructive Pulmonary Disease is a form of severe Acute Respiratory Failure (ARF) requiring Conventional Oxygen Therapy (COT) in the case of absence of acidosis or the application of Non-Invasive Ventilation (NIV) in case of respiratory acidosis. In the last decade, High Flow through Nasal Cannula (HFNC) has been increasingly used, mainly in patients with hypoxemic ARF. However, some studies were also published in AECOPD patients, and some evidence emerged. In this review, after describing the mechanism underlying potential clinical benefits, we analyzed the possible clinical application of HFNC to AECOPD patients. In the case of respiratory acidosis, the gold-standard treatment remains NIV, supported by strong evidence in favor. However, HFNC may be considered as an alternative to NIV if the latter fails for intolerance. HFNC should also be considered and preferred to COT at NIV breaks and weaning. Finally, HFNC should also be preferred to COT as first-line oxygen treatment in AECOPD patients without respiratory acidosis.

## 1. Introduction

Chronic Obstructive Pulmonary Disease (COPD) is a common disease characterized by persistent respiratory symptoms and airflow limitation [1]. Patients’ history is characterized by episodes of exacerbation with worsening respiratory symptoms, commonly precipitated by upper respiratory infection [1].

In case of severe exacerbation, COPD patients may develop an acute respiratory failure (ARF) of varying entities, sometimes requiring hospital admission due to the deterioration of the gas exchange. While only conventional oxygen therapy (COT) may be required in case of sole hypoxemia, respiratory acidosis and carbon dioxide (CO_2_) retention may ensue in 20% of patients because of an excessive respiratory workload over the respiratory muscles pump capacity [2]. In these latter cases, non-invasive ventilation (NIV) plays a major role. NIV has been shown to improve gas exchange, reduce breathing difficulty and the need for intubation and decrease hospital length of stay and mortality [2]. In particular, NIV is recommended for all those patients with ARF leading to acute or acute-on-chronic respiratory acidosis (pH ≤ 7.35), whereas there is no indication if patients encounter an acute exacerbation of COPD (AECOPD) and hypercapnia without acidosis [2]. Of note, up to 64% of AECOPD patients may fail NIV mainly due to worsened respiratory function, intolerance of the interface, cardiovascular instability and neurological deterioration [3]. In these cases, intubation is required, and invasive mechanical ventilation (iMV) is instituted [3].

The High Flow through Nasal Cannula (HFNC) was introduced in clinical practice, and its role is gaining more and more importance. Several studies investigated its application also in AECOPD patients for the treatment of the hypercapnic ARF [4,5]. 

After a brief explanation of the rationale and possible physiologic advantages of HFNC in AECOPD patients, we aim to provide a focus on their possible current clinical application in this population of patients.

## 2. Potential Advantages of HFNC in AECOPD Patients

HFNC delivers heated and humidified air–oxygen mixture to the patient, with an inspiratory fraction of oxygen (FiO_2_) ranging from 21 to 100% and a flow up to 60 L/min through a large bore nasal cannula [6,7]. HFNC has some potential advantages for AECOPD patients, which herein are discussed [7].

### 2.1. Heated and Humidified Gas Delivery

In healthy subjects, the upper respiratory tract humidifies the inspired room air to full saturation of water vapor (absolute humidity = 44 mg/L) and heats at 37 °C [8]. However, the administration of not conditioned medical gases, such as during COT or NIV, affects the ciliary motion, damages the respiratory tract epithelial cell and reduces the water content of the bronchial secretions [9,10,11]. This is of particular relevance in AECOPD patients, which are characterized by the production of copious secretions that need to be expectorated [4,5]. Indeed, in the case of accumulation of the secretions in the airways, the risk of hospital and ICU acquired infections strongly increases [11]. The use of active and heated humidification may reduce this risk by limiting the inflammatory response and bronchial epithelial cell damage [12] and by ameliorating the mucociliary clearance and cough effectiveness [4,5,13,14].

### 2.2. Anatomical Dead Space Washout

The volume of air located in the segments of the respiratory tract is responsible for conducting air from airways opening to the alveoli, without acting in the process of gas exchange is called dead space, and it includes the upper airways, trachea, bronchi and terminal bronchioles. 

In an upper airway model, HFNC was demonstrated to wash out the dead pharyngeal space from carbon dioxide (CO_2_) proportionally to the flow applied and the expiratory time [15]. In healthy subjects, the nasal cavity has a volume of about 40–50 mL, and it comprises at least 30% of the anatomical dead space in adults [16]. COPD patients are characterized by an incremented ratio between dead space and tidal volume [17]. The wash-out effect was advocated as one of the mechanisms to reduce the arterial partial pressure of CO_2_ (PaCO_2_) [18] and the respiratory drive after extubation [19] and at NIV discontinuation [20] as compared with COT.

### 2.3. “PEEP” Effect

HFNC also generates a small amount of positive end-expiratory pharyngeal pressure in healthy subjects [21,22,23] and in stable COPD or idiopathic pulmonary fibrosis [24]. The amount of generated positive pressure by HFNC depends on the flow delivered to the patient and the size of the nasal prong in relation to the nostrils [25,26]. The “PEEP” effect is produced by the expiratory resistance to the patient’s exhalation [21], which resembles the pursed-lip breathing pattern adopted by COPD patients [27]. This strategy diminishes the respiratory rate and prolongs the expiratory time, resulting in a reduction in the expiratory flow limitation and dynamic hyperinflation [27].

It is well known that the application of an external PEEP in COPD patients reduces the work of breathing in case of the presence of dynamic lung hyperinflation and intrinsic PEEP [28]. Together with the wash-out effect, the “PEEP” effect may explain the reduction in the respiratory muscle effort in both stable COPD patients [29] and in those recovering from an episode of exacerbation [19,20].

### 2.4. Provision of Stable Inspired Oxygen Fraction (FiO_2_)

Another potential advantage of HFNC is the delivery of stable inspired oxygen fraction (FiO_2_) to patients with ARF whenever the delivered flow exceeds the mean inspiratory peak flow of the patient [30]. Of note, in AECOPD patients, the mean inspiratory peak flow was reported to be around 70 L/min and exceed 60 L/min in about 70% of patients [31]. By delivering flow at around 60 L/min, HFNC guarantees a more stable FiO2 to AECOPD patients, as compared to COT through nasal prongs or Venturi masks.

### 2.5. Treatment Comfort

Among the most important determinants for treatment success, the comfort of the patient and the tolerance of the device play a major role during NIV [3,32]. The delivery of dry oxygen is perceived as uncomfortable and may generate pain related to mouth, throat and airways dryness; this is particularly true in critically ill patients [33]. 

Since the delivered gas admixture is heated and humidified, HFNC reduces this uncomfortable feeling as compared to both COT [20,30] and NIV [34]. In addition, nasal prongs are more tolerated than face masks for NIV, which may produce skin breakdown on the point of the pressure of the interfaces (i.e., forehead and nose) [34,35].

## 3. Clinical Application of HFNC in Exacerbated COPD Patients

According to the need for therapy, COPD exacerbation can be classified as mild, moderate and severe. A mild exacerbation is defined when the patient requires only treatment with short-acting beta-agonist bronchodilators (SABA), moderate if the hospitalization is required, in conjunction with SABA and/or corticosteroids therapy, severe when the exacerbation is associated with ARF [1]. The most important symptoms are dyspnea, increased sputum, cough and wheezing [1]. Within this spectrum of manifestation and degree of exacerbation severity, indications to oxygen and/or respiratory support vary.

In the case of sole hypoxemia, AECOPD patients require COT, whereas NIV is deemed if respiratory acidosis ensues. The recent guidelines strongly recommend the application of NIV whenever hypercapnic ARF with acidosis is present [2]. Moreover, a trial of NIV is also recommended if AECOPD patients would require iMV unless immediate deterioration occurs [2]. Of note, NIV was shown to improve gas exchange, reduce breathing difficulty and the need for intubation and decrease hospital length of stay and mortality [2].

While on one side, NIV provides these advantages, it is also affected by some drawbacks, such as patient–ventilator asynchrony, patient’s discomfort and intolerance to the treatment, leading to treatment failure [36,37,38,39,40,41,42,43]. Management of these issues becomes fundamental to avoid NIV failure, but it is not easy to achieve. For example, although patient–ventilator asynchrony can be partially managed by optimizing ventilator setting and/or modes of ventilation [36,37,38,39,40,41,42,43,44,45,46,47,48,49], the detection of asynchronous events is challenging if attempted by the sole ventilator waveform observation without the use of additional signals [50]. In addition, the patient’s discomfort and intolerance to the interface may be averted by adopting a rotating strategy and application of different interfaces, such as the helmet, possibly combined with specific ventilator settings [37,43,51].

In AECOPD patients, HFNC was demonstrated to reduce the retention of CO_2_ [52,53] and the activation of the diaphragm to a similar extent to NIV [20,54]. In addition, HFNC is well tolerated by patients [4,5]. Therefore, HFNC may have a potential role in the management of AECOPD patients, and several studies investigated the use of HFNC in this population as an alternative to NIV or to COT.

### 3.1. HFNC Settings in AECOPD Patients

Settings for HFNC are quite heterogeneous among all studies. Generally speaking, it would be preferred to set a flow between 35 and 60 L/min and titrate as much as tolerated by the patient. In addition, the temperature of the gas flow should be set between 34 and 37 °C, according to the patient’s tolerance. In the end, the FiO_2_ should be adjusted in order to obtain a SpO_2_ between 88 and 92% [55].

### 3.2. HFNC as an Alternative to NIV

Based on the aforementioned mechanisms, HFNC was used to test AECOPD patients as an alternative to NIV as first-line treatment in case of respiratory acidosis or after extubation.

The first applications in this sense were reported as case reports [56,57,58] or series [59]. In these reports, the alternative use of HFNC was deemed to be poor tolerance of the NIV interface [56,57,59] or had massive unmanageable air leaks [58], and it was successful with respect to both gas exchange and tolerance [56,57,58,59]. More recently, some prospective randomized controlled trials were published. 

Cong et al. randomized 168 AECOPD patients with respiratory acidosis to receive NIV or HFNC as first-line treatment [60]. Both treatments improved gas exchanges in a similar fashion after 12 h and 5 days of treatment. In addition, the time spent under respiratory support and the hospital length of stay was similar between treatment, although HFNC guaranteed fewer complications and it was more comfortable as compared to NIV [60].

Cortegiani et al. designed a multicenter randomized controlled trial to assess the noninferiority of HFNC compared to NIV with respect to the reduction in PaCO_2_ in AECOPD patients with mild-to-moderate respiratory acidosis [61]. The trial randomized 80 patients; HFNC was found to be non-inferior to NIV with respect to PaCO_2_. However, it should be mentioned that one-third of the patients in the HFNC group were switched to NIV within 6 h from randomization mainly because of lack of gas exchange improvement [62]. 

Doshi et al. conducted a subgroup analysis from a randomized controlled trial to compare HFNC and NIV in AECOPD patients with respect to gas exchange [63]. The authors reported that gas exchange, ICU and total length of stay and treatment failure (i.e., intubation rate and need to switch to other treatment) were similar between HFNC and NIV [63].

The recent European Respiratory Society Guidelines still suggest firstly attempting NIV in AECOPD patients since the existing evidence is large and strong; however, if NIV fails due to poor tolerance and gas exchange are not worsening, HFNC may be attempted [55].

HFNC was also compared to NIV after extubation of COPD patients recovering from an episode of exacerbation. Zhang et al. randomized all intubated AECOPD patients admitted in the ICU to receive NIV or HFNC at extubation [64]. The authors reported that HFNC reduced the ICU length of stay; however, no differences were recorded between HFNC and NIV in terms of gas exchange, 28 days reintubation rate and mortality [64]. In 42 AECOPD patients randomized to receive NIV or HFNC after extubation, Jing et al. also reported no differences between HFNC and NIV with regard to gas exchange, vital signs and some major clinical outcomes (i.e., the time spent under iMV, need for reintubation, ICU length of stay and cause of 28 days mortality) [34].

### 3.3. HFNC as an Alternative to COT

In AECOPD patients, HFNC was also compared to COT as first-line oxygen treatment in the absence of respiratory acidosis, at NIV discontinuation or after extubation.

In AECOPD patients without respiratory acidosis, Kim et al. firstly demonstrated that PaCO_2_ significantly decreased after 1 h of HFNC applied as first-line treatment [65]. In keeping with Kim et al. [65], Pilcher et al. also showed that the application of HFNC at 35 L/min reduced the transcutaneous CO_2_ tension in 24 exacerbated COPD patients, as opposed to COT via nasal prongs [52].

Our group randomized 30 COPD patients recovering from an episode of severe exacerbation to receive HFNC and COT during NIV breaks [20]. HFNC and COT guaranteed similar gas exchange, although the activation of the diaphragm and respiratory rate was significantly higher during COT, as compared to both HFNC and NIV. On the opposite, HFNC and NIV were similar. In a post hoc analysis, we also found that the need to reinstitute NIV at discontinuation was lower with HFNC (27%) as compared to COT (47%) [20].

Finally, Di Mussi et al. demonstrated randomized 14 COPD patients recovering from an exacerbation episode to receive HFNC or COT after extubation [19]. In this context, HFNC was superior to COT since it significantly reduces the work of breathing and the respiratory drive [19].

## 4. Discussion

The evidence supporting the use of HFNC is increasing in patients with hypoxemic ARF or after extubation, whereas data on AECOPD are still weak [55]. The recent guidelines suggest a trial of NIV prior to the use of HFNC in the case of AECOPD with respiratory acidosis [55]. Indeed, the literature recommends the application of NIV in AECOPD patients with respiratory acidosis [2], whereas the certainty of the evidence for mortality and intubation in favor of HFNC is low, mainly due to imprecision and heterogeneity among trials [55]. However, HFNC may have a role in this population. 

**Figure 1:**
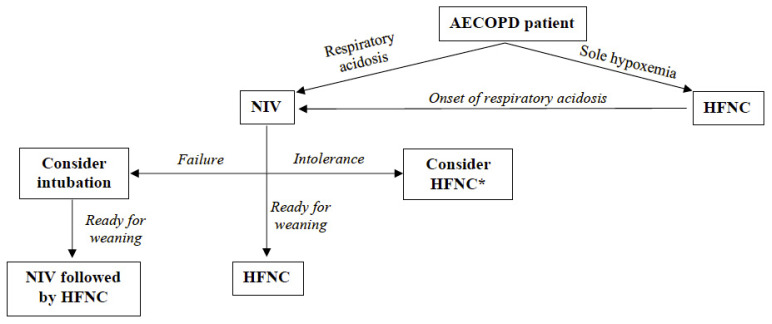
Flowchart of possible use of HFNC in AECOPD patients. * if clinical conditions and gas exchange are not deteriorating. AECOPD, Acute Exacerbation of Chronic Obstructive Pulmonary Disease; NIV, Non-Invasive Ventilation; HFNC, High Flow through Nasal Cannula.

Figure 1 proposes a clinical flowchart of possible uses of HFNC and approaches to AECOPD in varying settings and timing, based on the current literature. If an AECOPD patient is admitted to the hospital, an arterial blood gas analysis is required to define the presence of respiratory acidosis. If ARF is characterized by hypoxemia without respiratory acidosis, HFNC might be preferred over COT. Clinicians every time should consider that an exacerbation without acidosis may precipitate due to the increased resistive load (i.e., bronchospasm, increased secretion volume. If respiratory acidosis occurs, NIV must be instituted, and an NIV trial must be attempted. At this point, three scenarios may occur: (1) if gas exchange and/or clinical condition improve, the patient can be weaned applying HFNC at NIV interruption; (2) if the patient does not tolerate NIV and gas exchange and/or clinical condition do not deteriorate, HFNC may be used as an alternative to NIV; (3) if gas exchange and/or clinical condition further impair, the physician should consider indications for intubation and iMV. Finally, when an intubated AECOPD patient is ready for a weaning attempt from iMV, NIV is recommended at extubation to facilitate the weaning process and to prevent the occurrence of post-extubation respiratory failure. At NIV interruption, HFNC should be again preferred over COT.

NIV is affected by some drawbacks leading to patients’ intolerance and treatment failure. Factors associated with intolerance are multiple. NIV is commonly applied in pneumatically-triggered and cycled-off Pressure Support mode through a face mask [66]. The intolerance to the interface and poor patient–ventilator interaction and synchrony are major factors leading to NIV failure [36,39,41,43]. Some strategies to reduce these drawbacks are: (1) the application of interfaces such as the helmet [35,51,67,68,69], associated with a rotating strategy [40,70,71]; (2) the use of proportional modes of ventilation, i.e., the Neurally Adjusted Ventilatory Assist (NAVA) [47], with specific settings to improve pressurization and to trigger performance [37,41,43]. 

Despite these tricks, NIV may encounter failure due to poor comfort and patient tolerance. In this scenario, HFNC plays its role as an alternative treatment for AECOPD patients [55]. It should be strengthened that HFNC could be applied only in case of NIV intolerance [56,57,58,59], and if the gas exchange or clinical conditions are not worsening, otherwise intubation should be considered [2].

HFNC can also be used as an alternative to COT, either in AECOPD without respiratory acidosis or during NIV interruptions when the patients are recovering from the exacerbation episode. In these cases, HFNC has the advantage of being more comfortable than COT, to humidify the airways to facilitate expectoration of the mucus [20] and to wash out the dead pharyngeal space, reducing the respiratory drive [19,20]. A recent systematic review and meta-analysis further highlighted the physiological advantages of HFNC over COT in AECOPD. In particular, HFNC reduces the respiratory rate and effort as opposed to COT, although the evidence is low [72].

Currently, NIV is also suggested as a strategy to facilitate weaning from iMV in patients with hypercapnic respiratory failure [2]. The literature indicates that the use of NIV after extubation to facilitate weaning reduces mortality, the rate of weaning failure and the incidence of ventilator-associated pneumonia [2]. However, if NIV cannot be used or is problematic, HFNC may be used after extubation [19,34,64].

## 5. Conclusions

HFNC is a valuable tool in the management of AECOPD patients, as an alternative to NIV in case of intolerance, at NIV interruption, after extubation and as oxygen therapy instead of COT. Further large trials are required to strengthen the evidence for their application in this population.

## Data Availability

Not applicable.
